# Beta-1-Adrenergic Receptor Antibodies in Acute Coronary Syndrome: Is Less Sometimes More?

**DOI:** 10.3389/fcvm.2018.00170

**Published:** 2018-11-22

**Authors:** Diana Ernst, Christian Widera, Desiree Weiberg, Thorsten Derlin, Gerrit Ahrenstorf, Georgios Sogkas, Alexandra Jablonka, Reinhold E. Schmidt, Torsten Witte, Harald Heidecke, Gabriela Riemekasten

**Affiliations:** ^1^Department of Rheumatology and Immunology, Hanover Medical School, Hanover, Germany; ^2^Department of Cardiology, Carl von Ossietzky University Oldenburg, Oldenburg, Germany; ^3^Department of Nuclear Medicine, Hanover Medical School, Hanover, Germany; ^4^CellTrend GmbH, Luckenwalde, Germany; ^5^Department of Rheumatology, University of Schleswig-Holstein, Lübeck, Germany

**Keywords:** anti-beta-1-adrenergic receptor antibodies, biomarker, antibody, ACS, atherosclerosis

## Abstract

**Background:** Anti-beta-1-adrenergic receptor antibodies (anti-β_1_AR Ab) are associated with ischemic cardiomyopathies (ICM). Evidence continues to emerge supporting an autoimmune component to various cardiac diseases. This study compares anti-β_1_AR Ab concentrations in patients with different entities of acute coronary syndromes (ACS) to asymptomatic non-ACS patients with positron-emission computed tomography (PET/CT)-proven atherosclerosis, and healthy controls.

**Methods:** Serum anti-β_1_AR Ab IgG concentrations were measured in 212 ACS patients, 100 atherosclerosis patients, and 62 controls using ELISA. All ACS patients underwent coronary angiography. All 374 patients participating completed a structured questionnaire regarding traditional cardiovascular risk factors. ACS patients were followed up for 6 months.

**Results:** Patients with ACS exhibited lower anti-β_1_AR Ab levels compared to patients with atherosclerosis or healthy controls (both *p* < 0.001). No differences in the ab levels were evident between healthy controls and patients with atherosclerosis. In the ACS groups, lower concentrations were found in patients with ST-elevation myocardial infarction (STEMI) (0.67 μg/ml) compared to patients with angina pectoris (AP) and non-ST elevation myocardial infarction (NSTEMI) (both 0.76 μg/ml, *p* = 0.008). Anti-β_1_AR Ab levels ≤ 0.772 μg/ml were predictive for death and reinfarction (AUC 0.77, *p* = 0.006). No significant correlations between anti-β_1_AR Ab levels and atherosclerotic burden or traditional cardiovascular risk factors were identified.

**Conclusions:** Lower anti-β_1_AR Ab concentrations appear to characterize ACS phenotypes and could serve as diagnostic and prognostic markers independent from traditional risk factors for atheroscle. The prognostic predictive value of anti-β_1_AR Ab in ACS remains to be confirmed in larger studies.

## Introduction

Cardiovascular diseases (CVD) are common, accounting for approximately 17.3 million global deaths annually (1). Substantial evidence exists for numerous risk factors including dyslipidemia, smoking, arterial hypertension, diabetes, and abdominal obesity (2). In addition, several autoantibodies (ab) have been implicated in both CVD and atherosclerosis suggesting an autoimmune component in their complex pathophysiology atherosclerosis and CVD (3–6).

Numerous biomarkers have previously been reported in ACS, relating to both inflammation (CRP, Il-6, Il-10, Il-13) or myocardial damage [Troponin-T, Myoglobulin and Creatine Kinase-MB (CK-MB)] (7). Furthermore, prognostic markers such as Growth differentiation factor-15 (GDF-15) have emerged as promising predictors of post-STEMI mortality (8).

Anti-beta-1-adrenergic receptor antibodies (anti-β_1_AR Ab) were first identified in the 1980s suggesting their possible influence on cardiac anti-β_1_AR Ab activity and adenylate cyclase activity (9). They have been mainly reported in association with cardiomyopathy, heart failure (HF), and ischemic heart disease (IHD) (10, 11). The exact pathophysiological function of ß1AdrRab remains unclear and conflicting results exist about their effects on cardiac function: Nagatomo et al. showing favorable effects of IgG3 anti-β_1_AR Ab in HF (12, 13) whilst most other publications report a mainly negative influence of anti-β_1_AR Ab, especially in cardiomyopathy related to Chagas disease (14).

Anti-β_1_AR are highly expressed in the membranes of all three major cardiac cell types, i.e., myocytes, fibroblasts and endothelial cells (15). Their regulation plays a crucial role in cardiac function and morphology (16).

Anti-β_1_AR Ab belong to a group of autoantibodies against guanine nucleotide binding G-protein coupled receptors (GPCR). G proteins can stimulate or inhibit the enzyme adenylyl-cyclase, or activate phospholipase C. G protein-coupled receptor kinases (GRKs) play an important role in the regulation of adrenergic responses (15).

Anti-GPCR antibodies revealed their capability to initiate and propagate autoimmune diseases, as well as, their inherent functional properties in either activating or inhibiting intracellular signaling. As a result, they may be considered as both antagonistic or agonistic autoantibodies (17, 18).

The aim of this study was to measure anti-β_1_AR Ab in patients with confirmed acute coronary syndromes (ACS) and compare levels to those in both healthy controls and patients with PET/CT-proven atherosclerosis in the absence of an ACS.

## Methods

### Cohorts

All patients and participants gave verbal and written informed consent prior to study participation. The ethics committee of Hannover Medical School approved the study and the study protocol conforms to the ethical guidelines of the 1975 Declaration of Helsinki.

#### Acute coronary syndrome cohort

Between August 2007 and July 2011 unselected patients aged between 18 and 65 years admitted and subsequently diagnosed with ACS at our institution were included. To profile individual cardiac risk all patients completed a questionnaire assessing their past-history of hypertension, hypercholesterolemia, statin treatment, diabetes, tobacco exposure, as well as, previous vascular events including myocardial infarction and stroke. All patients provided a serum sample, which was subsequently tested for anti-β_1_AR Ab. Standard ACS diagnostics included temporal laboratory parameters, electrocardiogram (ECG), and coronary angiography. Laboratory tests included high-sensitivity C-reactive Protein (hsCRP), N-terminal pro-peptide brain natriuretic peptide (NT-pro-BNP), creatinine kinase, TroponinT (TnT), growth-differentiation factor 15 (GDF-15), total cholesterol, and LDL levels. Patient outcomes were followed up for at least 6 months after the index admission.

For sub-analysis, ACS patients were divided into three groups based on test findings: unstable angina pectoris (AP), non-ST elevation myocardial infarction (NSTEMI), and ST elevation myocardial infarction (STEMI). AP patients required at least 1 angiographically documented stenosis ≥70% in a major coronary artery.

Based on ST-ECG changes and level of TnT, using a decision threshold of 0.03 μg/L, STEMI or NSTEMI was diagnosed.

#### PET/CT cohort

Unselected adult patients attending for hybrid ^18^F-fluorodeoxyglucose (FDG) PET/CT scans at our institution between February 2015 and October 2015 were included on an a priori basis as described previously (3). Exclusion criteria were a history of vasculitis or other systemic inflammatory diseases, chemotherapy, or radiotherapy within the preceding 4 weeks or a current immunosuppressive therapy.

All participants completed the same structured questionnaire assessing their cardiac risks and provided a serum sample for testing anti-β_1_AR Ab. The questionnaire was completed under supervision from the same physician in all cases. Patient responses were subsequently validated by reviewing existing medical documentation.

In addition to their actual indication, all PET/CT scans were specifically reassessed by blinded physicians for evidence of atherosclerosis in 3 primary regions: carotid arteries, aorta, and ilio-femoral arteries. All studies were performed on a dedicated PET/CT system (Siemens Biograph mCT 128 Flow; Siemens Knoxville, TN) equipped with an extended field-of-view LSO PET component, a 128-slice spiral CT component, and a magnetically driven table optimized for continuous scanning. ^18^F-FDG was injected intravenously at a dose of 210 ± 16 MBq. After 60 min, imaging commenced with a single low-dose non-enhanced helical CT (120 kV, mA modulated, pitch 1.2, reconstructed axial slice thickness 5.0 mm) performed for PET attenuation correction. Whole-body PET images were then acquired in all patients using continuous bed motion at a speed of 1.1 mm/s for head, chest, and abdomen, and 2.1 mm/s for legs. Studies were reconstructed using time-of-flight and point-spread function TrueX information combined with an iterative algorithm (Ultra HD®, Siemens Healthcare; 2 iterations, 21 subsets, matrix 200; zoom 1.0; Gaussian filter of 2.0).

Images were analyzed with syngo.via software (Siemens Healthcare) on a dedicated workstation using segment-based analysis, in which major arteries were divided into the following 6 sites: right and left common carotid arteries, thoracic aorta, abdominal aorta, right and left iliac, as well as, right and left femoral arteries.

Calcified plaques (CP) were defined as high-density mural areas (attenuation >130 Hounsfield units). CT images were analyzed for CPs (19). Patients were categorized as having identificable CP (CT+), if at least one segment was bearing one calcified lesion, or as having CT negative results (CT–). In order to obtain a whole-body measure of calcified atherosclerotic burden on CT, all lesions within an individual patient were summed up to obtain the whole-body calcified plaque burden as described previously (3).

PET images were evaluated for focal radiotracer uptake in the arterial vessel walls (PET+). The localization of PET lesions was subsequently compared to CPs identified on PET/CT fusion images. Semi-quantitative analyses were then performed by calculating the maximum standardized uptake (SUV_peak_) by manually placing an individual region of interest (ROI) around the lesion on co-registered trans-axial PET/CT images. For the calculation of the arterial target-to-background ratio (TBR), the Standardized Uptake Value (SUV) of each arterial lesion was divided by the mean of blood-pool SUV (SUV_blood−pool_) derived from 3 circular ROIs placed in the mid lumen of the superior vena cava.

#### Control group

Sera from a group of verified healthy adult controls (Table 1), previously described in detail (20), were tested for anti-β_1_AR Ab. All participants had undergone screening with cardiac magnetic resonance imaging (cMRI) including dobutamine or adenosine stress tests, a 12-lead ECG, and physical examination without any pathological findings. All had returned normal laboratory results for serum creatinine, aspartate aminotransferase, alanine aminotransferase, thyroid-stimulating hormone, hemoglobin concentrations, leukocyte, platelet counts, oral glucose tolerance test, and N-terminal pro–B-type natriuretic peptide (NT-proBNP) levels. None of the participants were currently receiving medication and none had evidence of any conventional cardiovascular risk factors.

**Table 1:** Summarizing clinical demographics for participants in all groups.

	**Controls**		**PET/CT**		**ACS phenotype**					
					**AP**		**NSTEMI**		**STEMI**	
*N*	62		100		47		48		117	
Male, *N* (%)	36	(58)	44	(44)	38	(81)	40	(83)	104	(89)
Age, years	39	[27-47]	50	[41–58]	57	[49–64]	57	[48–61]	52	[41–58]
Never smoking, *N* (%)		–	44	(44)	1	(2)	7	(15)	17	(15)
Chest pain, hours		–	–		5.8	[1.8–20.0]	5.8	[2.4–34.3]	3.7	[2.9–9.0]
≤ 2 h pain, *N* (%)		–	–		14	(30)	10	(21)	16	(14)
>2 h pain, *N* (%)		–	–		32	(70)	35	(79)	98	(86)
Hypertension, *N* (%)		–	31	(31)	9	(19)	25	(52)	65	(56)
Diabetes, *N* (%)		–	3	(3)	6	(13)	10	(21)	11	(9)
Hyperlipidemia, *N* (%)		–	13	(13)	4	(9)	29	(60)	52	(44)
Prev MI, N (%)		–	5	(5)	19	(40)	8	(17)	15	(13)
1-year survival, N (%)		–	–		46	(98)	47	(98)	110	(94)
Anti-β_1_AR Ab μg/ml	1.08	[0.78–1.48]	1.06	[0.83–1.43]	0.76	[0.57–0.93]	0.76	[0.61–1.11]	0.71	[0.50–0.91]
Trop-T, ng/l					6.7	[4.2–15.9]	80.2	[23.5–503.5]	133.3	[39.1–710.9]
CK_max_, U/l					84	[68–152]	185	[94–391]	311	[150–777]
NT-BNP, ng/l				142.3	[55.8–260.0]	251.0	[74.2–251.0]	118.5	[56.5–540.2]
GDF-15 pg/ml					1369	[875–2094]	1399	[1141–1737]	1497	[1142–2130]
hsCRP, mg/l					1.79	[0.49–4.98]	2.63	[0.86–6.13]	2.28	[0.99–7–89]

**Figure 1:**
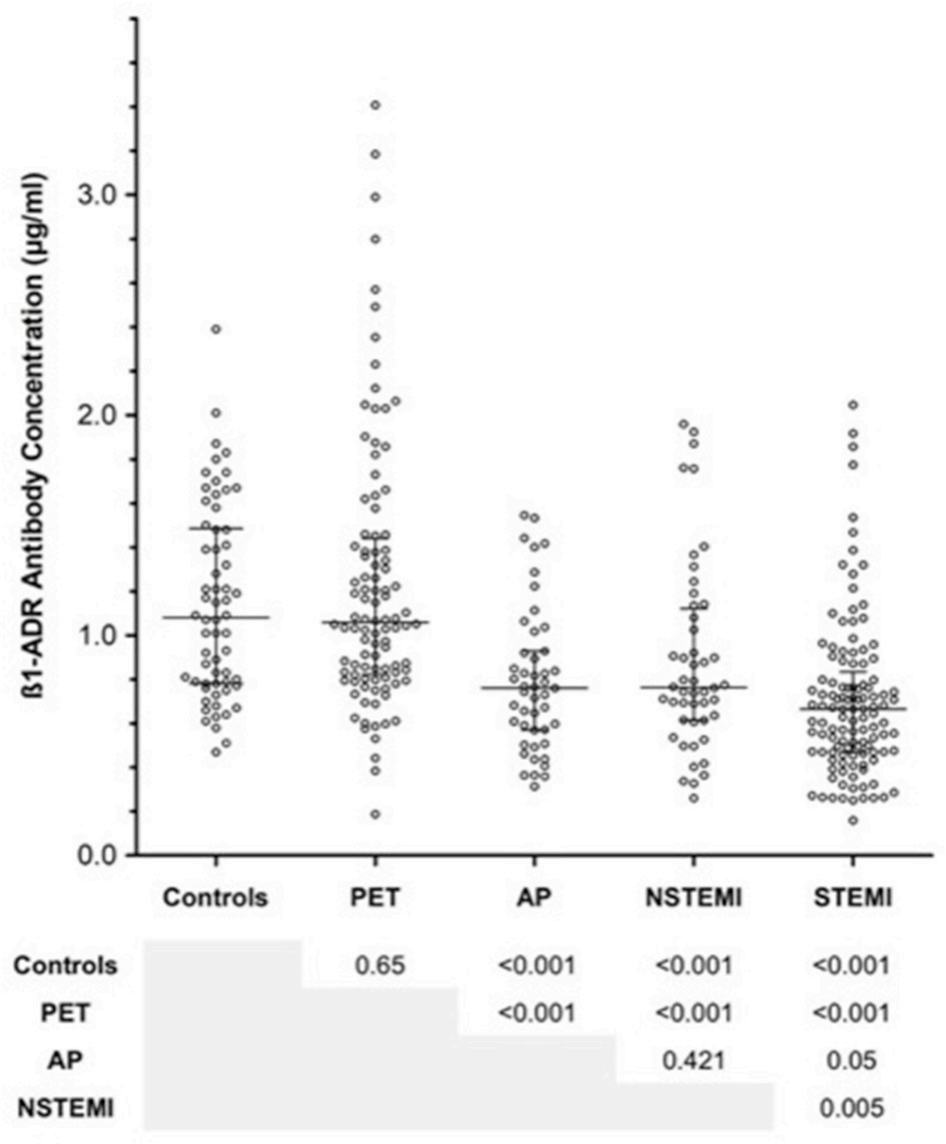
Summarizing the concentrations of anti-β_1_AR Ab within each of the groups, including the acute coronary syndrome (ACS) sub-groups. No differences were evident between the PET/CT group and healthy controls. By comparison, antibody concentrations among ACS groups were significantly lower than non-ACS patients. Within the ACS sub-groups, no differences were observed between angina pectoris (AP) and non-ST elevation myocardial infarction (NSTEMI). However, ST-elevation myocardial infarction (STEMI) patients exhibited lower levels of anti-β_1_AR Ab than both AP and STEMI patients. Error bars reflect median and interquartile range. *P*-values were obtained using Mann-Whitney *U*-test.

*Values are median [interquartile range] unless otherwise stated. All clinical variables demonstrated a highly significant difference (p < 0.001) across groups when tested using Kruskal-Wallis H-test. Controls were excluded from risk factor comparisons. Regarding ß1adrRab concentrations, these are compared in detail in Figure 1. ACS, acute coronary syndrome; PET/CT, positron emission tomography; AP, angina pectoris; NSTEMI, non-ST elevation myocardial infarction; STEMI, ST elevation myocardial infarction; Prev MI, previous myocardial infarction; anti-β_1_AR Ab, ßeta1 adrenergic receptor antibody; Trop-T, Troponin-T (laboratory reference value ≤ 15 ng/l); CK_max_, maximum creatinine kinase (laboratory reference value ≤ 168 U/l); B-NP, B naturetic peptide (laboratory reference value ≤ 146 ng/l); GDF-15, Growth differentiation factor; hsCRP, high-sensitivity C-reactive protein (laboratory reference value < 0.5 mg/l)*.

### Detection of anti-β_1_AR ab by ELISA

To quantify anti-β_1_AR Ab, a commercially available CE-certified ELISA kit (CellTrend GmbH, Luckenwalde, Germany) was used. Native membrane extracts from cell lines overexpressing human ß_1_AdrR were used to measure IgG via 7-transmembrane-ß1-Receptors in its assumed physiological conformation, as previously described (21, 22).

Microtiter 96-well polystyrene plates were coated with extracts from transfected Chinese Hamster Ovary cells overexpressing the human ß_1_AdrR. Conformational epitopes of the receptor were maintained by adding 1 mM calcium chloride to each buffer. Duplicate samples of a 1:100 serum dilution were incubated at 48°C for 2 h. After washing, plates were incubated for 60 min with a 1:20,000 dilution of horseradish peroxidase-labeled goat anti-human IgG (Jackson, Bar Harbor, ME, USA) for detection. In order to obtain a standard curve (Supplementary Figure 1), plates were incubated with a human monoclonal Antibody against ß_1_AdrR (ß_1_AdrR_Mab_). The ELISA was validated according to the FDA's “Guidance for industry: Bioanalytical method validation” (23). To standardize antibody concentrations, a standard curve was generated. Specifically, (a) 6250 ng/ml ß_1_AdrR_Mab_ for standard point 1, (b) 3125 ng/ml ß_1_AdrR_Mab_ for standard point 2, (c) 1563 ng/ml ß_1_AdrR_Mab_ for standard point 3, (d) 781 ng/ml ß_1_AdrR_Mab_ for standard point 4 (e) 391 ng/ml ß_1_AdrR_Mab_ for standard point 5 and (f) 195 ng/ml ß_1_AdrR_Mab_ for standard point 6. Then the optical density was determined. Each standard point was performed in duplicates. The detection threshold of ß_1_AdrRAbs was set at 100 ng/ml. All sera were coded and analyzed for ß_1_AdrRAbs assessment by individuals who had no information regarding the patients' characteristics.

### Statistics

Continuous variables were tested using the Shapiro-Wilk Normality Test. Where non-parametric distributions were identified, variables were tested using either the Mann Whitney *U*-test or Kruskal Wallis test depending upon the number of covariates under assessment. Otherwise, the Student's *T*-test or standard ANOVA were used. Categorical variables were similarly assessed using Chi^2^ test or Fisher's exact test. Correlations between continuous variables were equally assumed to be non-parametric and tested using Spearman's Rank Correlation Test. All *p-*values are 2-tailed, with 95% cut-off being the declared level of significance. Statistical analysis was performed using IBM SPSS Statistics for Macintosh, Version 23 (IBM Corp. Armonk, USA) and Prism 7 (GraphPad Software, La Jolla, USA).

## Results

### Patient demographics

In total 374 patients were included in the analysis: 212 (57%) ACS patients, 100 (27%) patients with overt atherosclerosis verified by PET/CT, and 62 (16%) controls. Basic demographic data are summarized in Table 1. Participants in the control group were significantly younger than those included in the other groups. To assess the influence of both age and gender on anti-β_1_AR Ab, a sub-analysis correlating both factors among all non-ACS patients was performed. Neither factor exhibited a significant correlation with antibody levels (*p* = 0.23 and *p* = 0.06, respectively). Among ACS patients, both collectively and within their respective subgroups, no significant difference in antibody concentration was evident between males and females. A strong male predominance was evident among all ACS sub-groups, which was not replicated in either the control or PET/CT sub-groups. Within the ACS group, the majority of participants (117/212; 55%) experienced a STEMI, with remainder being equally split among NSTEMI and troponin negative angina pectoris. Regarding duration of symptoms at presentation, no differences were evident between the AP and NSTEMI sub-groups (median 5.8 h, both groups). The median symptom duration among STEMI patients appeared shorter at 3.7 [2.9–9.0] h, but the inter-group differences did not achieve significance (*p* = 0.49). Compared to the AP (70%) and NSTEMI (79%) groups, a greater proportion of STEMI patients (86%) presented >2 h after symptom onset (*p* = 0.05).

Smoking, diabetes, hyperlipidemia, and previous myocardial infarction were more prevalent among ACS patients compared to PET/CT group, as well as, controls, who did not have any cardiac risk factors.

### Comparison of anti-β_1_AR ab between groups

**Figure 2:**
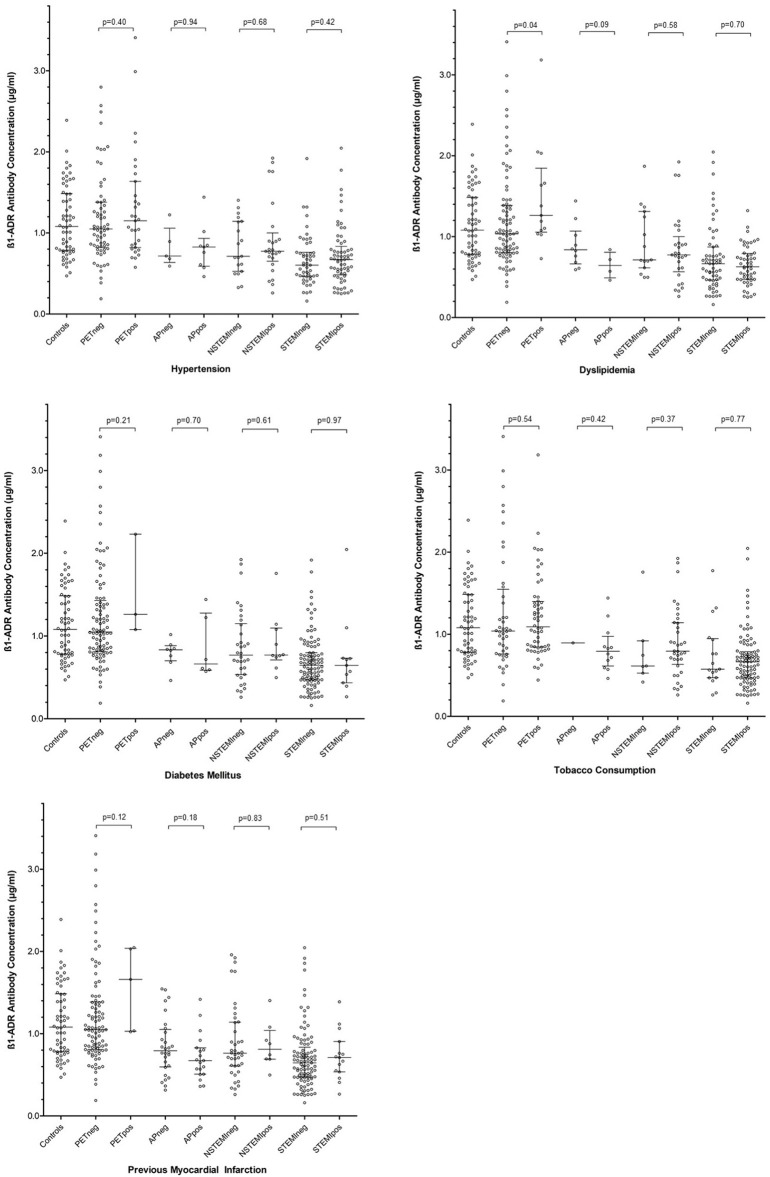
Summarizing the influence of individual cardiovascular risk factors on concentrations of anti-β_1_AR Ab within each patient group. Error bars represent median and interquartile ranges. *P-*values are 2-tailed and were calculated using the Mann Whitney *U*-test. PETneg, positron emission tomography group negative for the risk factor illustrated; PETpos, positron emission tomography group positive for the risk factor illustrated; APneg, angina pectoris group negative for the risk factor illustrated; APpos, angina pectoris group positive for the risk factor illustrated; NSTEMIneg, non-ST elevation myocardial infarction group negative for the risk factor illustrated; NSTEMIpos, non-ST elevation myocardial infarction group positive for the risk factor illustrated; STEMIneg, ST elevation myocardial infarction group negative for the risk factor illustrated; STEMIpos, ST elevation myocardial infarction group positive for the risk factor illustrated; ß1-ADR-Antibody, anti-ßeta1 adrenergic receptor antibody.

Antibodies against anti-β_1_AR Ab across all clinical groups are summarized in Figure 2. Although no differences were observed between the PET/CT group and much younger control groups (median 1.058 vs. 1.080 μg/ml; *p* = 0.65), a significant reduction in anti-β_1_AR Ab levels occurred in all ACS sub-groups with the lowest ab concentrations evident in the STEMI group. While no significant difference was evident between AP and NSTEMI patients (0.760 vs. 0.763 μg/ml; *p* = 0.42), STEMI patients demonstrated significantly lower concentrations compared to both AP and NSTEMI patients (median 0.665 μl/ml; AP vs. STEMI *p* = 0.05; NSTEMI vs. STEMI *p* = 0.005). After adjusting for surviers the median Ab was very similar among ACS/NSTEMI (0.762 vs. 0.763) whereas STEMI survivors continued to demonstrate significantly lower values (*p* = 0.01).

### Cardiac risk factors and anti-β_1_ar ab

Given the variable prevalence of traditional cardiovascular risk factors between clinical groups, the effect of individual risk factors within each group was assessed in isolation. The detailed analysis is summarized in Figure 2. With regards to known hypertension, diabetes mellitus, and tobacco consumption, none appeared to influence anti-β_1_AR Ab within any of the clinical groups. Contradictory weak trends regarding the role of dyslipidemia were observed in the PET/CT and AP groups. Among the 100 PET/CT patients, dyslipidemia was associated with a higher anti-β_1_AR Ab (median 1.263 vs. 1.034 μg/ml; *p* = 0.04) whereas in AP, patients with dyslipidemia trended toward lower concentrations (median 0.837 vs. 0.644 μg/ml *p* = 0.09). However, the numbers of patients involved in the latter were small (*n* = 14).

### Comparison of anti-β_1_AR ab with standard ACS biomarkers

To ascertain if anti-β_1_AR Ab corroborated traditional biomarkers of myocardial damage, ACS-associated inflammation, or myocardial dysfunction, Spearman's Rank correlations were constructed among ACS patients. A negative correlation was observed between anti-β_1_AR Ab and initial serum creatinine kinase (CK) level (*p* = 0.03). However; no significant correlations were observed when compared to peak measured creatinine kinase (*p* = 0.14), or serum Troponin-T at admission (*p* = 0.08). Similarly, no correlations between anti-β_1_AR Ab concentrations and the reported symptom duration were evident (Spearman's Rank correlation *p* = 0.89). With regard to the degree of cardiac dysfunction at presentation, no correlation to B-Natriuretic Peptide (*p* = 0.82) was evident. Similarly, neither GDF15 (*p* = 0.77) nor high-sensitivity CRP concentrations (*p* = 0.69) correlated with anti-β_1_AR Ab levels at admission.

All patients with re-infarction [*n* = 5 (8%)] and or death [*n* = 4 (6%)] within 6 months had anti-β_1_AR Ab concentrations below the median value. Collectively, the median anti-β_1_AR Ab concentration among these patients was 0.48 [0.41–0.61] μg/ml compared to 0.71 [0.51–0.92] μg/ml for the surviving ACS patients (*p* = 0.004). ROC analysis to determine anti-β_1_AR Ab concentration predictive of death or reinfarction returned a cut-off of ≤ 0.772 μg/ml (AUC 0.77; *p* = 0.006).

### Atherosclerotic burden does not influence anti-β_1_AR ab

As outlined previously, an individual cumulative atherosclerotic burden was calculated for each patient in the PET/CT sub-group based upon both SUV uptake and the number and size of calcified arterial lesions on the accompanying CT. Again, the Spearman's Rank correlation failed to reach significance (*p* = 0.27), suggesting that anti-β_1_AR Ab does not relate to atherosclerosis directly. This corroborates further the similar median concentrations observed between the PET/CT group and healthy controls, despite the large age gap observed.

### Anti-β_1_AR ab as a marker of acute coronary syndromes

**Figure 3:**
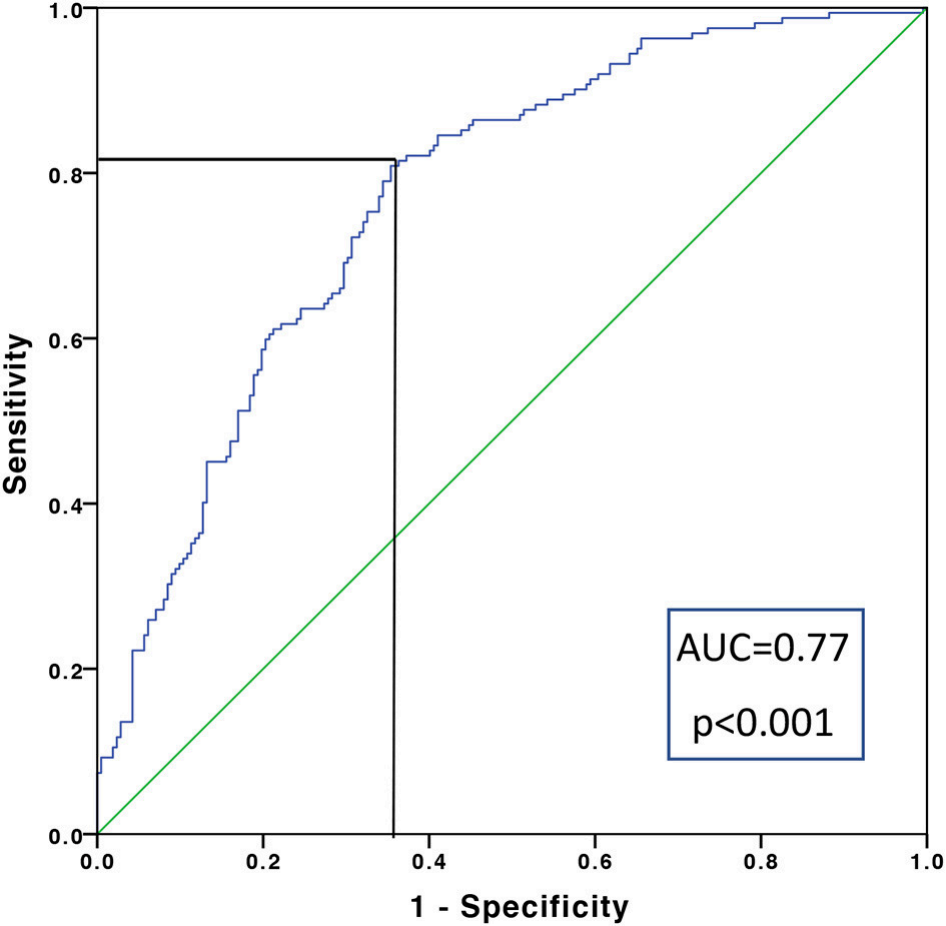
Receiver operating characteristic curve for detecting the presence or absence of an acute coronary syndrome across the entire cohort. Taking a sensitivity of 0.815 μg/ml and 1-specificity of 0.373 μg/ml, a cut-off in anti-β_1_AR Ab of 0.772 μg/ml was calculated.

Given the combined findings above, it was considered that a reduction in anti-β_1_AR Ab levels was in some way influential in the acute-phase of ACS. To explore the utility of anti-β_1_AR Ab in detecting ACS, a receiver operating characteristic analysis was performed. Taking 0.772 μg/ml as cut-off for anti-β_1_AR Ab concentrations provided the greatest accuracy in detecting ACS within the current cohort (Figure 3) and conferring a positive likelihood ratio of 33.7. Again, the reliability of this value beyond the current cohort cannot be ascertained and determination of the actual value would require large, controlled longitudinal data analyses.

## Discussion

This study reveals that patients experiencing ACS appear to have lower concentrations of anti-β_1_AR Ab compared to both healthy controls and non-ACS patients with atherosclerosis. Within the ACS sub-groups, reductions in antibody levels corresponded to the disease entities defined by dynamic ECG changes but not with conventional markers of muscle damage. The apparent correlation to initial CK was confounded by a marked variation in the time between symptom onset and presentation to hospital. This potentially introduces the need for temporal appraisal of anti-β_1_AR Ab during ACS evolution. Within the current cohort, reasonably robust discrimination between ACS phenotypes using anti-β_1_AR Ab concentrations was possible suggesting a potential diagnostic role for ACS and for the different ACS entities. Similarly, although the cohort was small, low anti-β_1_AR Ab concentrations show prognostic relevance for death or reinfarction within the subsequent 6 months. Collectively, the information elucidated suggests that low anti-β_1_AR Ab concentrations could reflect the acute clinical exacerbation rather than immunological or inflammatory responses to the current event. Given the characteristics revealed, it is similarly unsurprising that no relationships to traditional cardiovascular risk factors were observed.

Although little is currently known about the role of functional autoantibodies in the regulatory processes of cardiovascular disease, they continue to attract considerable interest (24, 25). Regarding anti-β_1_AR Ab specifically, it remains unclear if lower serum concentrations in ACS represent a risk factor for developing ACS, as the follow-up analyses suggested, or reflect consumption in response to acute injury at either an endothelial or myocardial level. However, low ß1AdrRab concentrations may be a valuable marker for ACS.

Previous studies have suggested that anti-β_1_AR Ab may bind to ß1-adrenergic receptors on cardiomyocytes, preventing further ischemic damage by antagonizing catecholaminergic processes (12, 13). In line with this, lower concentrations of anti-β_1_AR Ab were associated with recovery of left ventricular function (10).

Others have confirmed the prognostic relevance of low anti-β_1_AR Ab on both hospitalization and mortality in cardiac failure (13). Undoubtedly more research is needed to further characterize the exact function of ß1AdrRab in ACS. However, low levels of antibodies against the chemokine receptors CXCR3 and CXCR4 were also shown to be predictive for deterioration of progressive lung fibrosis (26), whereas high levels of antibodies against the endothelin receptor type-A were predictive for PAH and mortality (27) suggesting that both higher and lower ab levels against G protein coupled receptor could be of diagnostic and prognostic relevance.

Regarding the underlying pathophysiological processes, current evidence suggests that anti-β_1_AR Ab binding has various intracellular signaling effects. In DCM cohorts, anti-β_1_AR Ab exhibited agonistic effects, triggering cAMP-induced catecholaminergic mechanisms in cardiomyocytes, whereas in healthy controls it triggered receptor changes that did not lead to cAMP stimulation (28). It currently remains unclear which cofactors influence anti-β_1_AR Ab action and how exactly this relates to the pathogenesis of cardiomyopathies or ACS.

Our results provide further insight into these processes by suggesting that an event-related decrease in anti-β_1_AR Ab in ACS patients, given that levels in non-ACS patients with known atherosclerosis demonstrated similar concentrations to healthy controls. One plausible explanation would be some form of consumption or binding as a response to injury, in which an agonistic anti-β_1_AR Ab effect on cardiomyocytes contributing to the known positive ino- and chronotropic event response would result (29). Such responses could explain the progressively lower concentrations seen in STEMI vs. NSTEMI and AP (*p* = 0.008). Intriguingly, all of patients—admittedly a small number—experiencing re-infarction or death within 90 days, all possessed anti-β_1_AR Ab concentrations significantly lower than the remaining ACS patients, with ROC analysis returning a reasonably robust (AUC 0.77; *p* = 0.006) cut-off of 0.772 μg/ml within the current cohort exhibiting prognostic relevance. It should however be clearly reiterated, that the numbers involved here are small and both verification and validation are needed in larger suitable cohorts.

Several possible mechanisms could account for this. With increasing myocardial damage, repair mechanisms lead to antibody binding demands outstripping supply, leading to falling serum levels and inadequate antagonism of ino- and chronotropic processes, leading to further ischemic damage. Alternatively, increased anti-β_1_AR Ab binding may result in some form of paradoxical agonistic response, pathologically increasing catecholaminergic responses and exacerbating myocardial damage (30, 31). It is known that expression of β_1_AR Ab changes at different stages of myocardial ischemia, being up-regulated in ACS and down regulated in chronic ischemic heart failure (30, 31). These mechanisms may explain partial the quantitative findings of anti-β_1_AR Ab.

Intriguingly, anti-β_1_AR Ab were not associated with the presence of atherosclerosis itself. In accordance with these findings, traditional cardiovascular risk factors, which are determinants of the development of atherosclerotic lesions (32), were also not associated with anti-β_1_AR Ab concentrations, further supporting the assumption that the association of anti-β_1_AR Ab with ACS is not linked to atherosclerotic pathology in the arterial wall.

Independent from these pathophysiological mechanisms, our findings provide preliminary evidence that measuring anti-β_1_AR Ab concentrations may be useful in either diagnosis or risk stratifying ACS. A better understanding of the relationship with Troponin is certainly needed, but the current findings suggest that they do not share the same origin. A further attraction would be the utility of anti-β_1_AR Ab concentrations, particularly in patients where Troponin T is unreliable, such as in those with renal impairment.

### Study limitations

The data presented is derived from a relatively small cohort and should be considered as preliminary findings in need of validation in a larger appropriately designed prospective cohort. The current study is limited both by its retrospective nature and the use of a priori groups. The lack of longitudinal data and limited patient follow-up prevents any evaluation of subsequent ab concentrations. With regards to the ACS group, levels were checked on admission samples. It is equally conceivable that differences in initial management, particularly among STEMI patients, may in some way have influenced the serum values. Likewise, for patients with prior ACS in both the ACS and PET/CT groups, no ACS free interval was applied that could have influenced findings. Furthermore, no echocardiographic functional data was used either in determining the extent of myocardial dysfunction in the ACS cohort or excluding the possibility of DCM in either the ACS or PET/CT groups.

## Conclusion

Anti-β_1_AR Ab concentrations were significantly reduced in patients experiencing ACS, compared to apparently healthy controls, as well as, patients with proven atherosclerosis. The ACS entities correlated with the antibody concentrations and particularly low concentrations may have prognostic relevance. Further research is required to understand the pathophysiological processes governing anti-β_1_AR Ab levels in ACS. Similarly, the prognostic impact needs to be confirmed in a larger study.

## Author contributions

All authors take responsibility for all aspects of the reliability and freedom from bias of the data presented and their discussed interpretation. Contribution to the manuscript. DE conception and design of the study, acquisition of data, analysis, and interpretation of data, drafting the article, and final approval of the version to be submitted. CW, DW, GA, AJ, GS, and TD acquisition of data, or analysis and interpretation of data, revising critically for important intellectual content and final approval of the version to be submitted. HH acquisition of data, revising critically for important intellectual content and final approval of the version to be submitted. RS interpretation of data, revising critically for important intellectual content and final approval of the version to be submitted. GR and TW conception and design of the study, interpretation of data, revising critically for important intellectual content and final approval of the version to be submitted.

### Conflict of interest statement

HH is the owner of CellTrend. Neither HH nor the employees of CellTrend had access to clinical data. The remaining authors declare that the research was conducted in the absence of any commercial or financial relationships that could be construed as a potential conflict of interest.
