# Supplementary material for: Beta-1-Adrenergic Receptor Antibodies in Acute Coronary Syndrome: Is Less Sometimes More?
Source: Front Cardiovasc Med. 2018 Nov 22;5:170. doi: 10.3389/fcvm.2018.00170 (PMC6305491; doi:10.3389/fcvm.2018.00170)
Supplement: Supplementary file 1 [file Data_Sheet_1.docx]

Suppl. Fig. X1: Standard curve of the anti-β_1_AR Ab ELISA
